# *Leptospira* Seroprevalence and Risk Factors in Health Centre Patients in Hoima District, Western Uganda

**DOI:** 10.1371/journal.pntd.0004858

**Published:** 2016-08-03

**Authors:** Anou Dreyfus, Jonathan W. Dyal, Raewynne Pearson, Clovice Kankya, Charles Kajura, Lordrick Alinaitwe, Steven Kakooza, Katharine M. Pelican, Dominic A. Travis, Michael Mahero, David R. Boulware, Lawrence Mugisha

**Affiliations:** 1 Section of Epidemiology, Vetsuisse Faculty, University of Zurich, Zurich, Switzerland; 2 Section of Emergency Medicine, Baylor College of Medicine, Houston, United States of America; 3 Massey University, Palmerston North, New Zealand; 4 College of Veterinary Medicine, Animal Resources & Biosecurity, Makerere University, Kampala, Uganda; 5 Hoima District Local Government, Hoima, Uganda; 6 Central Diagnostic Laboratory, College of Veterinary Medicine, Animal Resources & Biosecurity, Makerere University, Kampala, Uganda; 7 Department of Veterinary Population Medicine, College of Veterinary Medicine, University of Minnesota, Minneapolis, United States of America; 8 Department of Medicine, University of Minnesota, Minneapolis, United States of America; 9 Conservation & Ecosystem Health Alliance (CEHA), Kampala, Uganda; Institut Pasteur, FRANCE

## Abstract

**Background:**

The burden of human leptospirosis in Uganda is unknown. We estimated the seroprevalence of *Leptospira* antibodies, probable acute/recent leptospirosis, and risk factors for seropositivity in humans in rural Western Uganda.

**Methodology and Principal Findings:**

359 non-pregnant adults visiting the Kikuube and Kigorobya Health Centers were sequentially recruited during March and April 2014. A health history survey and serum were collected from consented participants. Overall, 69% reported having fever in the past year, with 49% reporting malaria, 14% malaria relapse, 6% typhoid fever, 3% brucellosis, and 0% leptospirosis. We tested sera by microscopic agglutination test (MAT) against eight *Leptospira* serovars representing seven serogroups. *Leptospira* seroprevalence was 35% (126/359; 95%CI 30.2–40.3%) defined as MAT titer ≥ 1:100 for any serovar. The highest prevalence was against *L*. *borgpetersenii* Nigeria (serogroup Pyrogenes) at 19.8% (71/359; 95%CI 15.9–24.4%). The prevalence of probable recent leptospirosis (MAT titer ≥1:800) was 1.9% (95%CI 0.9–4.2%) and uniquely related to serovar Nigeria (serogroup Pyrogenes). Probable recent leptospirosis was associated with having self-reported malaria within the past year (p = 0.048). Higher risk activities included skinning cattle (n = 6) with 12.3 higher odds (95%CI 1.4–108.6; p = 0.024) of *Leptospira* seropositivity compared with those who had not. Participants living in close proximity to monkeys (n = 229) had 1.92 higher odds (95%CI 1.2–3.1; p = 0.009) of seropositivity compared with participants without monkeys nearby.

**Conclusions/Significance:**

The 35% prevalence of *Leptospira* antibodies suggests that exposure to leptospirosis is common in rural Uganda, in particular the Nigeria serovar (Pyrogenes serogroup). Leptospirosis should be a diagnostic consideration in febrile illness and “smear-negative malaria” in rural East Africa.

## Author Summary

Leptospirosis is an emerging zoonotic disease caused by bacteria of the genus *Leptospira*. Despite its relatively common frequency, mild/moderate leptospirosis often goes unrecognized, due to its usually non-specific symptoms of fever, vomiting, and malaise. Knowledge of leptospirosis in Uganda is limited, and the disease may often be misdiagnosed as malaria. This study sought to define the percentage of healthcare seeking Ugandans in rural Western Uganda who have antibodies to *Leptospira* in their blood, suggesting prior exposure. We found 35% of study participants had antibodies to at least one *Leptospira* serovar, predominantly *L*. *borgpetersenii* sv Nigeria representing the Pyrogenes serogroup (20% of all participants). Individuals with increased odds of having antibodies to leptospires included participants who skinned cattle and those who reported monkeys near their home. Individuals who self-reported recent diagnosis of malaria were more likely to have leptospirosis antibodies. Antibodies to leptospirosis are not lifelong, typically lasting a few years. The high 35% seroprevalence suggests there is ongoing exposure. Further studies are needed to understand the burden of leptospirosis in rural Africa, the risk factors associated with exposure, and the public health opportunities to mitigate leptospirosis.

## Introduction

Leptospirosis is a zoonotic bacterial disease with a worldwide distribution that is endemic in subtropical and tropical countries. Transmission occurs through exposure to urine or aborted tissues of infected animals, either through direct contact with carrier animals or contact with contaminated water or soil [1]. *Leptospira* is a Genus of spirochetes that comprise 20 species and almost 300 different serovars (sv). They have a large range of mammalian hosts which carry specifically adapted serovars in their renal tubules and excrete them in the environment for months or years. Humans are considered accidental hosts who most likely do not transmit the bacteria [1–3]. Infection patterns vary depending on the climate and rainfall, and the socio-economic, occupational, or recreational activities which bring a susceptible host into contact with infected water or animals. In tropical regions, leptospirosis outbreaks in animals and humans often occur after flooding [4].

Human incidence of leptospirosis is commonly underreported due to low awareness of the disease, lack of surveillance systems in place, nonspecific clinical symptoms, and the complexity of diagnosis [4,5]. The World Health Organization (WHO) Leptospirosis Epidemiology Reference Group (LERG) estimated in a 2015 systematic literature review that the annual incidence was 1.03 million cases (95%CI, 305,000–1,750,000) and 58,900 deaths (95%CI, 23,800–95,800) due to leptospirosis worldwide [6]. East Africa had an estimated annual incidence of 25.6 (95%CI 9.3–43.3) per 100,000 population [6].

Studies on leptospirosis in Uganda have only been published in animals. In 2011, Millan et al. [7] sampled 105 dogs around three national parks using the Microscopic Agglutination test (MAT) and found 27% (95%CI, 19–36) seropositivity; across six serovars, most frequently Icterohaemorragiae and Canicola [7]. In 2014, Atherstone found a 29% seropositivity in cattle (n = 92) and 42% seropositivity in buffaloes (n = 92) for *Leptospira* Hardjo serovar when using an ELISA [8].

Elsewhere in East Africa, a few studies on *Leptospira* prevalence in humans and animals have been published. A recently published systematic literature review on leptospirosis in Africa reported a prevalence of acute human leptospirosis ranging from 2.3% to 19.8% in 11 studies in hospital patients with acute febrile illness and compatible symptoms [9]. A study conducted in Tanzania in 2013 estimated 75–102 clinical leptospirosis cases per 100,000 population [10]. In two studies of Tanzanian cattle in 2011 and 2014, a 30% seroprevalence was found (n = 1758) using the MAT [11,12]. Assenga et al. reported a seroprevalence of 29% in buffaloes (n = 38), 20% in rodents (n = 207), and 30% in humans (n = 267) [12]. The most prevalent serovar was Hardjo in humans (16%) and cattle (18%), but in rodents was Australis (19%). These results point to a human-cattle transmission pathway, which is plausible in the agro-pastoral environment of Tanzania [12].

Although cross-sectional seroprevalence studies mainly indicate past exposure to leptospires, persons with high titers may have recent acute disease or recent recurrent exposure. Sero-conversion may serve as an imperfect, proxy measure of incidence. Biggs et al. demonstrated 33% of 870 acute febrile illness patients had antibodies against *Leptospira* serovar in Moshi, Tanzania in 2007–2008 [13]. Further, 8.8% of 870 had seroconverted with a greater than four-fold increase in serum MAT titer on convalescent testing, consistent with “confirmed leptospirosis”. An additional 3.6% had titers of ≥1:800 in one tested sample, and therefore suited the case definition of “probable recent leptospirosis” [13]. Among their laboratory confirmed cases of leptospirosis, 44% had previously received the clinical diagnosis of malaria.

Given these foundational studies and the similar eco-systems and agro-pastoral activities between Uganda and Tanzania, leptospirosis may present an unrecognized disease burden in Uganda, particularly when misdiagnosed as malaria or another febrile illness. This study’s objectives were to estimate the seroprevalence of *Leptospira* antibodies and probable recent leptospirosis in humans in rural western Uganda. Secondary objectives included analysis of risk factors for seropositivity against specific serovars and for probable recent leptospirosis.

### Description of the Study Environment

Hoima District in western Uganda presents unique challenges regarding diagnosis and management of leptospirosis. The district lies on the eastern coast of Lake Albert, the northwestern most of the African Great Lakes. Hoima contains a widely varied ecology, with protected conservation areas, mixed-use pastoral farm lands, coastal fishing villages, and urbanized towns. The climate is tropical, with average rainfall of 1270 mm/year, average temperatures above 21°C and two rainy seasons from March-May and August-November which can bring intermittent flooding [14]. Malaria is holoendemic and most commonly diagnosed by microscopy, with greater than 80% of children under the age of ten infected [15]. As a result of these high levels of parasitemia, febrile illnesses may be routinely diagnosed as malaria, obscuring the true burden of other infectious pathogens like leptospirosis. Over a quarter of the district’s 550,000 inhabitants live in poverty [14]. Occupations focus predominantly around subsistence agriculture characterized by mixed farming and pastoralism (i.e. livestock). Novel trends show increasing land area devoted to rice farming that requires periodic flooding and recent oil and gas extraction [16,17]. The district also contains the UNHCR refugee settlement at Kyangwali sub-county, where >20,000 refugees from Democratic Republic of Congo are located [16]. These mixed communities, land use patterns, and ecologies create a complex milieu allowing zoonotic disease transmission. Hoima’s community Health Centres serve this heterogeneous patient population.

## Methods

### Study Design, Data Collection and Management

Study participants were recruited at the Kikuube and Kigorobya Health Center IV’s within Hoima District during March and April 2014. The Health Centre IV’s provide service to ~100,000 Ugandans each. Every non-pregnant adult aged ≥ 18 years who presented to the health center, either as a patient or as a caregiver, was invited to participate. Common reasons for seeking health care at these centers included: obstetric complications, occupational trauma, and acute febrile illnesses. Demographic interviews were conducted by clinical officers, and serum samples were collected from both field sites on a daily basis. Samples were centrifuged and then stored in -20°C freezers at the Hoima Regional Referral Hospital. Sera were transported on ice to the Ministry of Agriculture, Animal Industry, and Fisheries in Entebbe, and stored at -80°C prior to testing.

### Participant Survey

Survey data were collected in four major areas (S1 Text). First, information on demographics including age, gender, education, religion, profession, sub-county of residence, and duration of residence in Hoima was collected. Second, past medical history assessed for prior diagnosis with febrile diseases such as malaria, typhoid, brucellosis, leptospirosis, and hemorrhagic fevers. Additional questions assessed recent history of fever within the past year, whether the participant had seen a physician and the outcome of any treatment for febrile illness. Third, animal contact was evaluated using quantity of animals owned, animal product consumption during the last month, involvement in cattle or beef processing within the past two weeks, and the presence or absence of wildlife, such as rats or monkeys, near the home. Finally, questions regarding the participant’s domestic environment included the fabrication material used to construct their home and their major sources of drinking water (Table 1). Data on acute clinical status, such as fever of the patient at the Health Centre, was not collected.

**Table 1:** Frequencies of exposure variables, *Leptospira* seropositivity (any serovar) by exposure variable categories and the bivariable association (odds ratio, OR) between an exposure variable and seropositivity against any serovar (Any) and sv Nigeria (Ni) in 359 participants from Hoima District, Uganda.

Exposure Variable	Categories	N (%)	Seropositivity N (%)	OR (Any)	95% CI (Any)	OR (Ni)	95% CI (Ni)
Health Center	Kikuube	181 (50)	64 (35)	ref		ref	
	Kigorobya	178 (50)	54 (30)	0.8	0.5–1.2	0.9	0.5–1.5
Sex	Women	263 (73)	85 (32)	ref		ref	
	Men	96 (27)	33 (34)	1.1	0.7–1.8	1.1	0.6–1.9
Age	18–29	155 (44)	54 (35)	ref	ref	ref	
	30–39	91 (26)	26 (28)	0.7	0.4–1.3	0.4	0.2–2.1
	40–49	59 (17)	19 (32)	0.9	0.5–1.7	1.1	0.5–2.2
	>50	47 (13)	16 (34)	1.0	0.5–1.9	0.7	0.3–1.8
Education	None	63 (18)	19 (16)	ref		ref	
	Primary	219 (61)	74 (63)	1.2	0.6–2.2	1.2	0.6–2.4
	Secondary	68 (19)	21 (18)	1	0.5–2.2	0.7	0.3–1.7
	Post-Secondary	9 (3)	4 (3)	1.9	0.4–7.7	1.2	0.2–6.6
Religion	Christian	335 (93)	107 (32)	ref		ref	
	Muslim	14 (4)	6 (43)	1.6	0.5–4.6	-	-
	Traditional	10 (3)	3 (30)	0.9	0.2–3.5	1.0	0.2–4.6
Occupation	Farming^1^	292 (81)	94 (32)	0.9	0.5–1.5	1.7	0.8–3.7
	Domestic Work^1^	138 (38)	43 (31)	0.9	0.6–1.47	1.1	0.6–1.8
Time in Hoima (yrs)	<1	16 (4)	7 (6)	ref		ref	
	1—<3	12 (3)	6 (5)	1.3	0.3–5.8	0.6	0.1–4.0
	3—<5	7 (2)	2 (2)	0.5	0.1–3.5	1	-
	5—<10	4 (1)	0 (0)	1	-	1	-
	10+	320 (89)	103 (87)	0.6	0.2–1.7	0.8	0.2–2.5
Livestock Contact	Herding^1^	70 (20)	27 (38)	1.4	0.8–2.3	1.3	0.7–2.4
	Milking^1^	25 (7)	5 (20)	0.5	0.2–1.3	0.8	0.2–2.3
	Birthing^1^	9 (2.5)	0 (0)	-	-	-	-
	Slaughtering^1^	6 (1.7)	3 (50)	2.1	0.4–10.4	2.1	0.4–11.4
	**Skinning**^1^	6 (1.7)	5 (83)	**10.6**	**1.2–91.9**	4.2	0.8–21.2
	Butchering^1^	3 (1)	3 (100)	-	-	2.0	0.2–22.8
High Risk Activity 1^2^	No	351 (98)	3 (38)	ref		ref	
	Yes	8 (2)	5 (63)	3.5	0.8–15.0	2.5	0.6–10.7
High Risk Activity 2^3^	No	285 (80)	45 (61)	ref		ref	
	Yes	74 (21)	29 (39)	1.4	0.8–2.4	1.3	0.7–2.4
Other Animal Contact	**Monkeys**^1^	229 (64)	86 (37)	**1.8**	**1.1–3.0**	**2.1**	**1.2–3.7**
	Baboons^1^	58 (16)	20 (34)	1.1	0.6–2.0	0.7	0.3–1.5
	Chimpanzees^1^	43 (12)	15 (35)	1.1	0.6–2.1	0.9	0.4–2.1
	Rats^1^	346 (96)	115 (33)	1.7	0.4–6.1	1.4	0.3–6.3
	Bats^1^	180 (50)	57 (32)	0.9	0.6–1.48	1.0	0.6–1.6
	Deer^1^	8 (2)	4 (50)	2.1	0.5–8.5	2.5	0.6–10.7
Type of Housing	Mud, Thatch	171 (48)	53 (45)	ref		ref	
	Mud, Cow Dung, Thatch	2 (0.5)	0 (0)	1	-	1	-
	Mud, Iron	101 (28)	35 (30)	1.2	0.7–2.0	1.3	0.7–2.3
	Concrete, Iron	85 (24)	30 (25)	1.2	0.7–2.1	1	0.5–2.0
Past Medical History ≤ 1 year	Fever^1^	249 (69)	78 (31)	0.8	0.5–1.3	0.9	0.5–1.6
	Malaria^1^	175 (49)	52 (30)	0.8	0.5–1.2	0.7	0.4–1.2
	Typhoid^1^	20 (6)	7 (35)	1.1	0.4–2.8	1.8	0.7–4.9
	Brucellosis^1^	12 (3)	0 (0)	-	-	-	-
Water Sources	Piped water in home^1^	36 (10)	13 (36)	1.2	0.6–2.4	1.2	0.5–2.7
	Bore Hole^1^	154 (43)	52 (34)	1.1	0.7–1.7	1.0	0.6–1.6
	Well^1^	134 (37)	42 (31)	0.9	0.6–1.4	0.9	0.5–1.5
	Public Tap^1^	20 (6)	5 (25)	0.7	0.2–1.9	0.7	0.2–2.5
	Surface Water^1^	45 (13)	13 (29)	0.8	0.4–1.6	1.0	0.5–2.2
Sub-counties	Kigorobya	171 (48)	52 (30)	ref		ref	
	Bugambe^4^	6 (2)	2 (33)	1.1	0.4–2.6	1.4	0.5–3.7
	Kabwoya^4^	7 (2)	3 (43)	1.1	0.4–2.6	1.4	0.5–3.7
	Kitoba^4^	2 (0.6)	1 (50)	1.1	0.4–2.6	1.4	0.5–3.7
	Kyabigambire^4^	1 (0.3)	0 (0)	1.1	0.4–2.6	1.4	0.5–3.7
	Kyangwali^4^	1 (0.3)	0 (0)	1.1	0.4–2.6	1.4	0.5–3.7
	Buhimba	14 (4)	7 (50)	2.3	0.8–6.8	2.4	0.7–7.7
	Kiziranfumbi	149 (42)	51 (34)	1.2	0.7–1.9	1.0	0.6–1.8

^1^The reference category consists of all other observations, which do not belong to the category (i.e. farming yes/no)

^2^ Slaughtering, skinning or butchering cattle

^3^ Slaughtering, skinning, butchering, milking or birthing cattle

^4^ these sub-counties were summarized in one category, **bold** = statistically significant (p≤0.05)

### Serological Testing

The MAT is the reference test for distinguishing among leptospirosis serovars and giving valuable information on past exposures [2]. Due to the absence of MAT laboratory capacity in Uganda and the lack of isolated and cultured local strains, *Leptospira* serovars and corresponding antisera were imported from the OIE Reference Laboratory Royal Tropical Institute (KIT), Holland. A panel of eleven serovars from ten different serogroups (sg) was chosen based on the recommendations of experts from KIT, represented in Table 2. These serovars were selected to represent a wide variety of serogroups with minimal crossreactivity, most of which had been isolated from other parts of Africa. *Leptospira Kirschneri* sv Soikoine, *Leptospira borgpetersenii* sv Kenya and *Leptospira borgpetersenii* sv Nona were not tested, as these antigens did not grow well during the testing phase or were contaminated and not regarded fit for MAT testing. MATs were performed in the framework of a capacity building course run by the authors Pearson and Dreyfus in conjunction with authors Alinaitwe and Kakooza. In an effort to partner equally with our Ugandan colleagues, development of MAT capacity in country was considered an essential component of the project.

**Table 2:** Serovar panel, seroprevalence of *Leptospira* serovars and serogroups by Microscopic Agglutination Test (titer ≥1:100) among 359 humans sampled in Hoima, Uganda.

*Leptospira Serovar*	*Serogroup*	*N positive*	Prevalence*	*95% CI*
*L*. *borgpetersenii* sv Nigeria	Pyrogenes	71	19.8%	15.9–24.4
*L*. *borgpetersenii* sv Hardjobovis**	Sejroe	20	5.6%	3.5–8.6
*L*. *interrogans* sv Wolffi**	Sejroe	19	5.3%	3.3–8.3
*L*. *kirschneri* sv Butembo	Autumnalis	8	2.2%	1.0–4.5
*L*. *interrogans* sv Bratislava	Australis	7	1.9%	0.9–4.2
*L*. *kirschneri* sv Grippotyphosa	Grippotyphosa	1	0.3%	0.0–1.5
*L*. *interrogans* sv Icterohemorrhagiae	Icterohemorrhagiae	0	0.0%	0.0–1.0
*L*. *biflexa* sv Patoc	Semaranga	0	0.0%	0.0–1.0
*L*. *kirschneri* Sokoine***	Icterohemorrhagiae	-	-	-
*L*. *borgpetersenii* Kenya***	Ballum	-	-	-
*L*. *borgpetersenii* Nona***	Hebdomadis	-	-	-
*Any* Leptospira *serovar*	*Any positivity*	*126*	*35*.*0*%	*30*.*2–40*.*3*

* This prevalence is an apparent prevalence, as the Microscopic Agglutination Test is not 100% sensitive and specific.

** Cross-reactions likely between serovars. Seven persons (1.9%) had MAT titers ≥1:800 consistent with probable recent leptospirosis.

*** Did not grow properly and were not tested.

The serum aliquots were transferred to microtiter plates and stored at -80°C. The MAT measured serum antibodies at doubling two-fold dilutions starting at 1:25 dilution up through 1:3200, as described previously [2]. Given the absence of information on prevalent serovars in Uganda, serovars were considered representative of their serogroup, and within serogroup cross-reactivity was not excluded.

### Data Analysis

A seropositive case was defined as a MAT titer of ≥1:100 against any serovar. A probable recent leptospirosis case was defined as a MAT titer of ≥1:800 [13,18]. The outcomes of interest were the prevalence of study participants who were seropositive against each specific serovar, seropositive against any serovar, or seropositive with probable recent leptospirosis.

Past medical history was based on self-reported survey information. No biometric or serological data were available to confirm self-reported data. Questionnaire information and serologic test results were entered into Microsoft Access and analyzed using Excel and Stata 10 (StataCorp, College Station, TX, USA). Data deposited in the Dryad repository: http://dx.doi.org/10.5061/dryad.6ns6p [19]. Exploratory data analysis was conducted to evaluate crude associations using 2x2 tables, histograms and summary measures. To improve power in the risk factor analysis, two composite variables were created to identify groups with persons with intensive contact with livestock and potential exposure to livestock associated *Leptospira* serovars. The first composite variable included persons involved in slaughtering, skinning and butchering cattle, while the second also included exposure to cattle milking and birthing.

A sample size of 278 enabled a 95% confidence interval (95% CI) within a precision of ±5% under the assumption that the true prevalence was 20% [20]. To detect an odds ratio of 2.5 with 80% power, a type I error of 0.05, prevalence of 9% in the exposed group, and an exposed to non-exposed ratio of one-third, the required sample size was 280 study participants.

The association between the outcomes and exposure variables listed in Table 1 was analyzed in two steps. Initial analysis included bivariable comparison of individual exposure variables with outcomes by chi-square tests or logistic regression, followed by a multivariable logistic regression. A manual forward and backward selection method was used to evaluate the association between exposure and confounding variables with the outcome. Exposure variables were entered in the model if the bivariable p-value was ≤0.2 or if they represented biologically plausible risk or confounding factors for the outcome and were kept in the model if the Likelihood Ratio Test was statistically significant (p≤0.05).

### Ethics Statement

All procedures involving human subjects were approved by the institutional review boards of the University of Minnesota, USA and Joint Clinical Research Centre, Uganda and the Uganda National Council of Science and Technology (UNCST). Potential participants were informed of the study during their healthcare encounter. After their routine healthcare visit, each person was given the opportunity to provide written informed consent.

## Results

### Study Population

A total of 359 participants provided informed consent, were interviewed, and had serum collected. Of those approached for participation, approximately 50% consented to participate. Participants originated from eight of 13 sub-counties in Hoima District of western Uganda, although 89% (320/359) came from the subcounties immediately surrounding each Health Centre. 71% of participants were women, and 69% (246/259) were <40 years of age. 81% participated in farming as either a primary or secondary occupation, with a minority involved with livestock on a daily basis, such as milking (7%), birthing (2.5%), slaughtering (1.7%), skinning (1.7%) and in butchering (1%) (Table 1).

### Past Medical History and Disease

Over their lifetime, 87% (n = 312) of 359 study participants had self-reported diagnoses of malaria, 16% (n = 56) typhoid, 4% (n = 16), brucellosis, and 1% (n = 4) reported a diagnosis of tuberculosis. No participants reported a diagnosis of leptospirosis. For illnesses within the past one year, 49% of participants reported a malaria diagnosis over the past year. Of participants with malaria diagnoses, 49 participants (20%) noted having had a relapse of fever after initial malaria treatment. Overall, 69% (n = 249) reported a history of fever within the past year, and of those who reported a fever, 70% (175/249) received a clinical diagnosis of malaria. For other illnesses, 8% (n = 20) self-reported typhoid enteric fever, and 5% (n = 12) self-reported a brucellosis diagnosis in the past year.

Regarding other zoonotic infections within the communities, 16% (n = 58) of participants reported to have known someone affected by brucellosis and 15% (n = 54) by rabies. No one reported leptospirosis in the community.

### *Leptospira* Seroprevalence and Antibody Titers

126 study participants (35.0%, 95%CI, 30.2–40.3%) were seropositive against any of the eight serovars. The diversity of responses against the different serovars, representing different serogroups is listed in Table 2. The most frequent reactivity to a serovar was to sv. Nigeria sg Pyrogenes at 19.8% (95% CI 15.9–24.4%, n = 71) which was statistically significantly higher than the prevalence of other serovars (p<0.0001) **(**Table 2). The second highest prevalence was caused by the sg Sejroe represented by sv Hardjobovis and sv Wolffii with 5.6% (95%CI 3.5–8.6) and 5.3% (95%CI 3.3–8.3), respectively. A very low or nonexistent prevalence was found against sv Bratislava sg Australis with 1.9% (95%CI 0.9–4.2), against sv & sg Grippotyphosa with 0.1% (95%CI 0.0–0.1), and sv & sg Icterohemorrhagiae and sv Patoc sg Semaranga with both 0.0% (95%CI 0.0–1.0).

The seroprevalence did not differ between the sub-counties, but the individual sub-county samples sizes were very small **(**S1 Fig). Furthermore, the seroprevalence did not differ by age group with 35% (54/155) of those aged 18–29 years, 29% (26/91) aged 30–39 years, 32% (19/59) aged 40–49, and 34% (16/47) aged ≥50 years being seropositive (P = 0.78) (Table 1).

**Fig 1:**
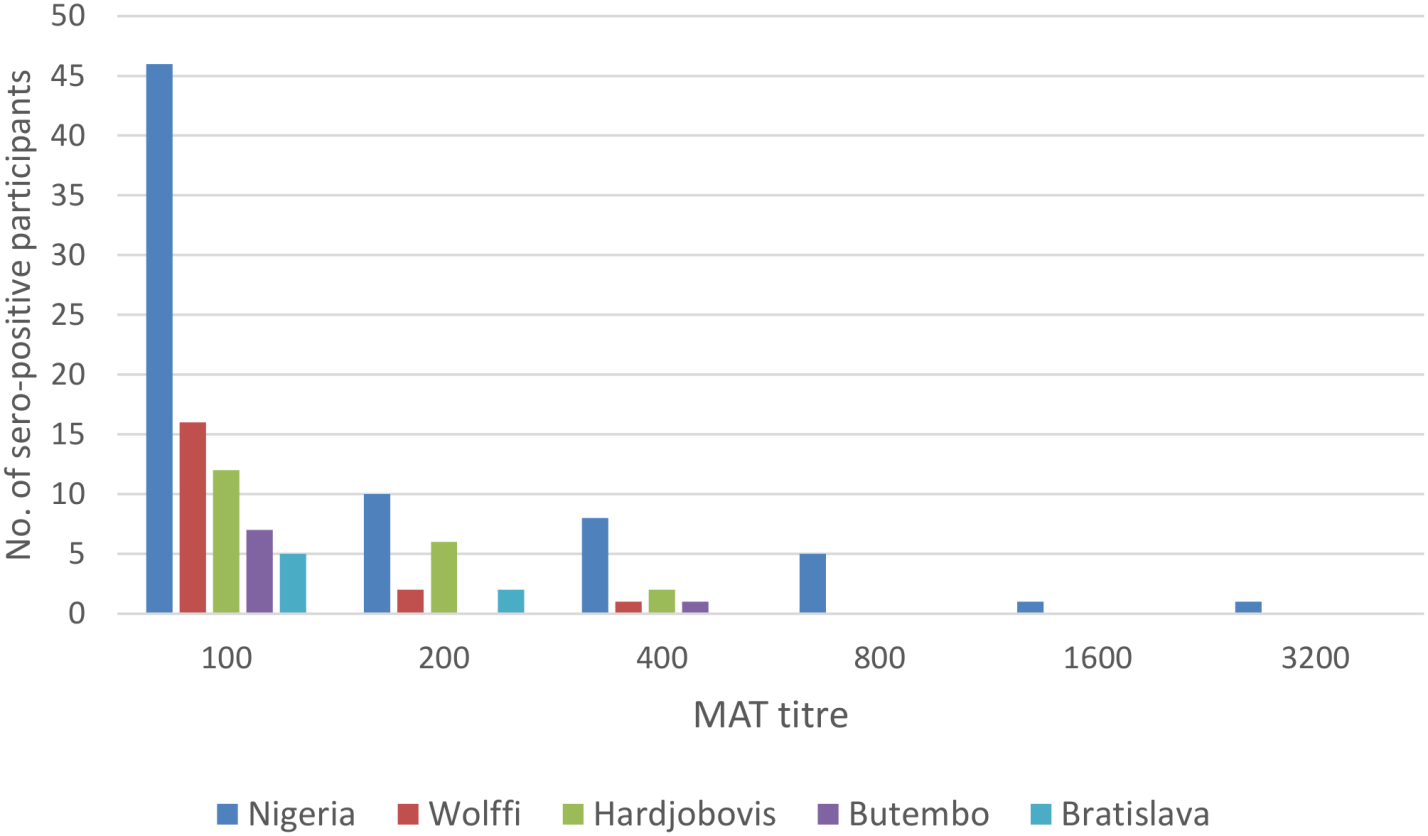
Frequency histogram showing the number of seropositive sera of patients (n = 359) at each MAT titer to different serovars.

Antibody titers against any serovar ranged from zero to 1:3200. The seven participants with titers ≥1:800 had solely antibodies against sv Nigeria (Fig 1). Therefore, the prevalence of probable recent leptospirosis was 1.9% (95% CI 0.9–4.2%) and was uniquely related to sv Nigeria. Eight (6.4%) of 125 seropositive participants had either been exposed to multiple serovars and/or their sera cross-reacted in the MAT. There were double exposures (or cross-reactions) between Nigeria and Wolffi (n = 1), Nigeria and Butembo (n = 1), Nigeria and Bratislava (n = 2), Nigeria and Hardjobovis (n = 2). Further, between Wolffi and Hardjo (n = 1) and Bratislava and Butembo (n = 1).

### Risk Factors for Probable Recent Leptospirosis and Seropositivity

All exposure variables (“risk factors”) listed in Table 1, including demographic, past medical history, and occupational/behavioral exposures were tested for a statistically significant association (p≤0.05) with the outcome “probable recent leptospirosis” by bivariable and multivariable analysis. Having been diagnosed with malaria in the past year was statistically significantly associated with being a case of probable recent leptospirosis (P = 0.048) in the chi-square test. However, in multivariable analysis, the association lost its statistical significance (OR 6.5, 95% CI 0.8–5, P = 0.085). All other health history risk factors, including having had fever, a fever that recurred after treatment, brucellosis, or typhoid in the past year, were not associated with probable recent leptospirosis in either analysis.

We further assessed risk factors listed in Table 1 for seropositivity against sv Nigeria, sv Hardjobovis, sv Wolffi, sv Butembo and sv Bratislava and for seropositivity against any of the eight serovars, by bivariable (Table 1) and multivariable analysis (Table 3). In the bivariable analysis, six participants who reported having skinned cattle in the two weeks prior to their blood sample had an odds ratio (OR) of 10.6 (95% CI 1.2–91.9, P = 0.032) for being seropositive against any serovar compared to participants who had not skinned cattle. People who reported monkeys living near their home had 2.1 (95% CI 1.2–3.7 P = 0.018) and 1.8 (95% CI 1.1–3.0 P = 0.013) times the odds of seropositivity against sv Nigeria and any serovar, respectively, compared to persons without monkeys nearby.

**Table 3:** Multivariable logistic regression: significant effects on seroprevalence of *Leptospira interrogans* Bratislava, *L*. *borgpetersenii* Nigeria and of any serovar listed in Table 2 among 359 humans sampled in Hoima, Uganda.

Outcome/Model	Risk Factor	Odds Ratio	95% CI	P-Value
Positivity against sv Bratislava	Skinning	9.88	1.01–96.04	0.048
Positivity against sv Nigeria	Contact with monkeys	2.05	1.13–3.71	0.018
Positivity against any *Leptospira* sv	Contact with monkeys	1.92	1.18–3.13	0.009
Positivity against any *Leptospira* sv	Skinning	12.3	1.4–108.6	0.024

The statistically significant risk factors in the multivariable analysis are listed in Table 3. Persons involved in skinning cattle had 9.8 (95% CI 1.0–96.0 P = 0.048) times the odds of seropositivity against sv Bratislava and 12.28 (95% CI 1.39–108.58 P = 0.024) times the odds of seropositivity against any serovar, once adjusted for the effect of having monkeys near the home. Individuals with monkeys near their home had 2.05 (95% CI 1.1–3.7 P = 0.018) and 1.92 (95% CI 1.2–3.1 P = 0.009) times the odds of seropositivity against sv Nigeria and any serovar respectively, once adjusted for the effect of skinning. Other variables tested, including the “livestock-contact” composite variables, ownership of livestock, consumption of animal products, wildlife exposures other than monkeys around the home, and frequency of forest visits were not significantly associated with seropositivity at the individual serovar or all serovar levels in the bivariable and multivariable analysis. Similarly, no association was found between housing materials or drinking water origin and *Leptospira* seropositivity.

## Discussion

We found 35% prevalence of serum antibodies against eight *Leptospira* serovars in humans in rural western Uganda. We further detected a seroprevalence of 20% against sv Nigeria sg Pyrogenes, which was the most frequent serovar or serogroup for exposure. Historically, sv Nigeria was isolated from bovine kidneys in Nigeria [21]. The high prevalence of antibodies against sv Nigeria raises concern for a bovine-human transmission pathway in western rural Uganda that deserves further examination. Despite their lower seroprevalence, the presence of antibodies against cattle-associated serovars Wolffi (5%) and Hardjobovis (6%) of the sg Sejroe also strengthen the cattle transmission hypothesis. [22–24].

In addition to the high overall seroprevalence, the prevalence of probable recent leptospirosis was 1.9%. The 1.9% of individuals with titers reflective of probable recent leptospirosis further emphasizes the potential public health relevance of serogroup Pyrogenes in Uganda. If the MAT cut-off for probable leptospirosis was lowered to a titer of ≥1:400 [25], which has been used in other studies, 5.6% (95% CI, 3.5–8.6%) of participants would fit the case definition of probable recent leptospirosis. In the bivariable analysis, probable recent leptospirosis was statistically significantly associated with the person having had malaria in the past year. One possible explanation for this association is the oft-lamented concern that leptospirosis may frequently be misdiagnosed as malaria. However this association should be considered cautiously, as fever and past medical history were purely self-reported variables, and may have significant inherent error and recall bias.

Had all participants specifically come to the health centers due to acute febrile illness, the prevalence of probable recent leptospirosis might have been higher, given that Biggs et al. [13] found that 9% of patients had confirmed leptospirosis who were admitted for acute febrile illness at two health centers in northern Tanzania. Although such clinical inclusion criteria were not used in our study due to ethical concerns, our estimates were more representative of the general population instead of a selected sub-sample of persons presenting only with febrile illness. However, some serovars of the species *L*. *borgpetersenii* have been shown to cause primarily asymptomatic infection in humans, making further characterization of sv Nigeria essential to understanding its importance in this community.

Of similar importance is the 0% seroprevalence to *L*. *interrogans* sv. Icterohemorrhagiae, traditionally associated with severe clinical infection. The ecology of different serovars has not been described for the study region, but possible explanations include that this serovar may not be present in this region, or that it is less likely to be found in relatively healthy, outpatient clinic visitors. This emphasizes the importance of further study of leptospirosis in clinically ill patients presenting with undifferentiated febrile disease.

Skinning of cattle was associated with seropositivity. Skinning is a biologically plausible risk factor for *Leptospira* seropositivity, as contact with contaminated urine is possible during the slaughter process. Why skinning and not the actual slaughter of cattle was associated is unclear, but may be due to a higher duration of exposure during the more intricate skinning process as opposed to the relatively brief exposure when cattle are killed. Similarly, skinning has been found to be the strongest risk factor for seroconversion in sheep slaughtering abattoirs in New Zealand [26]. The low sample size (n = 6) led to limited power to analyze risk factors and may be responsible for either artificially exaggerating the association with skinning or minimizing the association with other high risk behaviors. Given the consistency of these results with other studies, overall the risk factor analysis supports the bovine-human transmission hypothesis.

The association between having monkeys living near the home and seropositivity for leptospirosis is more ambiguous. These individuals may live in more remote areas with greater wildlife contact in general, thus giving them more exposure to wildlife classically associated with leptospirosis. The possibility of a more direct role for monkeys in the transmission of leptospires to humans in Uganda is not established here but could also be further explored. Leptospirosis is commonly associated with contact to rodents, however the ubiquitous exposure to rats limited the ability of the study to assess the role of rodent exposures.

The study design and the sampling approach have limitations which may affect generalizability. First, the participants of this cross-sectional study were a convenience sample of those who attended two health clinics for a variety of health problems. Although each Health Centre IV has a theoretical catchment area that covers half of Hoima District, in practice the large majority of patients came from the subcounties immediately surrounding each Health Centre. As a result, the more rural parts of Hoima, including the Kyangwali refugee camp, were highly underrepresented. Furthermore, the study population included only those ≥18 years old and women were overrepresented (71%), whereas in general the population of Hoima is 50% female. In rural areas, women were expected to have a lower *Leptospira* seroprevalence than men, due to differences in occupational exposures (i.e. high-risk livestock activities), but the overall seroprevalences in women (32%) and men (34%) were similar. Persons working in livestock were also under-represented, and more systematic population sampling may have revealed an even higher seroprevalence.

Alternatively, the seroprevalence may have been overestimated, as this was a predominantly outpatient clinic population and patients were more likely to be ill compared to the population at large. Since many of the participants had fever symptoms in the past year (70%), it is possible *Leptospira* seroprevalence was higher in the study population than in the general population. However, fever is a very common symptom, especially in rural Africa; hence it is plausible that 70% of the general population will have experienced a fever episode within one year.

The limited sampling period may also have affected the seroprevalence. March-May is traditionally a rainy season within Hoima, which could potentially increase the rates of acute leptospirosis. However, the season was drier than usual, and there were no episodes of flooding in the town center or health centers. Furthermore, while such immediate weather conditions might be reasonably blamed for acute, high titer cases; overall seropositivity for exposure should be less affected. Hence, there were many potential reasons for the study to have underestimated or overestimated community-level seroprevalence.

The definition of the variables involving contact with cattle (including skinning) were not ideal, as the exposure time was set for the last two weeks prior to the interview. The time span may have been too short for some participants to seroconvert in cases of exposure to *Leptospira* through animal contact. However, most people who endorsed having had contact with cattle within the two weeks prior to the interview will most likely also have had similar prior contact, as skinning or butchering are usually regular activities and antibodies may persist between several months and years [2,27]. Others may have had more remote exposures to these activities that were not captured by the survey. Finally, some of the analyzed exposures occurred rarely, leading to low power for the risk factor analysis (Table 1).

Since laboratory capacity for the MAT did not exist in Uganda, antigens and antisera were imported from the WHO reference laboratory in Holland, and the size of the serovar panel was limited. The chosen panel was not large enough to cover all the common serogroups, and may not encompass all local strains. Hence, the overall prevalence may have been underestimated in this study. Since testing was targeted towards past exposure to leptospires and not acute disease, a MAT sensitivity of 88% and specificity of 98% can be assumed [25]. Therefore, the tested prevalence is an “apparent seroprevalence” and will most likely be slightly underestimated.

An undisputed limitation is the lack of clinical data during the sampling phase. It would have been highly informative to analyze the association between febrile illness and high leptospirosis titers. Due to ethical concerns with collecting clinical data without the ability to immediately diagnose (diagnostic capacity for MAT or PCR was unavailable in Uganda during the sampling period) or treat individual patients, data on clinical status such as the presence of fever, subsequent symptoms or symptom duration was not collected. However, as a result of the capacity building component of this study, laboratory diagnosis of leptospirosis will be available to support future clinical studies.

This initial study on leptospirosis in Uganda raises several research questions of interest for future studies. We recommend further exploration of the “bovine-human transmission pathway” by testing sera (MAT) and urine/kidneys (PCR) of bovines and sera of humans working in their proximity, either in farming/pastoral communities or in abattoirs. Additional testing of rat kidneys for the presence of leptospires may help evaluate the significance of the bovine-human transmission pathway relative to classically described murine-human transmission. In order to estimate the burden of leptospirosis in Uganda and the clinical importance of prevalent serovars, a study in patients with acute febrile illness would also be useful. A case-control study within this study population could assess risk factors in their community, such as location, flooding, contact with different animal species and occupational activities. Yet, before launching a large surveillance study of specific serovars for a new region/country, one consideration might be first sampling abattoirs as likely hotspots of exposure. In testing abattoir workers’ serum for an extensive range of serovars, one might determine which serovars are circulating in a community and then perform targeted testing of acute febrile illness patients using a less expansive, less costly panel. Working in such hotspots may also facilitate isolation of actual pathogenic leptospires.

This is to our knowledge the first article reporting on the prevalence of antibodies against *Leptospira* serovars in humans in Uganda. The 35% prevalence of antibodies to *Leptospira* suggests frequent exposure to this pathogen, in particular the Nigeria serovar of the Pyrogenes serogroup. Given this exposure, leptospirosis may have a greater impact on the health of this population than previously recognized. Further research is needed to understand the public health impact of leptospirosis in Uganda.

## Supporting Information

S1 Fig Seroprevalence estimates (%) and 95% confidence intervals of *Leptospira* serovars from 359 participants in Hoima District, Uganda. (JPG)

S1 Text Study forms. (PDF)

S1 Checklist STROBE checklist. (DOC)
