# Supplementary figures and images for: Leptospira Seroprevalence and Risk Factors in Health Centre Patients in Hoima District, Western Uganda
Source: PLoS Negl Trop Dis. 2016 Aug 3;10(8):e0004858. doi: 10.1371/journal.pntd.0004858 (PMC4972303; doi:10.1371/journal.pntd.0004858)

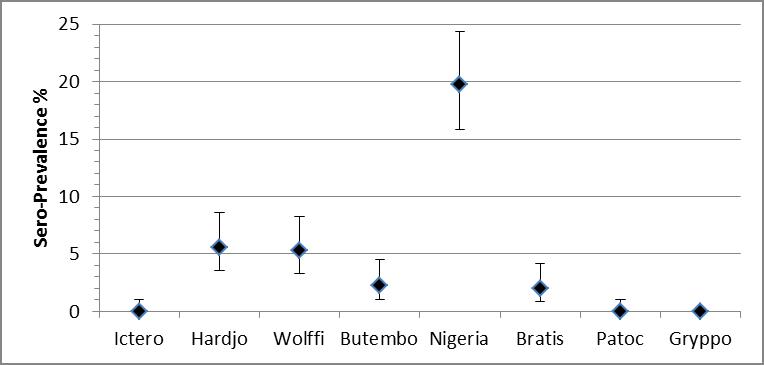

Supplement: S1 Fig — (JPG) [file pntd.0004858.s001.jpg]
