# Supplementary material for: Leptospira Seroprevalence and Risk Factors in Health Centre Patients in Hoima District, Western Uganda
Source: PLoS Negl Trop Dis. 2016 Aug 3;10(8):e0004858. doi: 10.1371/journal.pntd.0004858 (PMC4972303; doi:10.1371/journal.pntd.0004858)
Supplement: S1 Text — (PDF) [file pntd.0004858.s002.pdf]

Participant Procedure for ZDSS

1. Morning Announcement of Study and Discussion in Waiting Room
2. Patient is brought to Clinical Officer
3. Clinical Officer discusses ZDSS Study and storage of samples
4. Participant signs Informed Consent for Participation
5. Participant signs Sample Storage Consent
6. Clinical Officer Administers Risk Survey
7. Clinical Officer gives Patient the Consents and Survey
8. Clinic Staff bring Patient to the Laboratory
9. Patient gives Lab Technician their Consents and Survey
10. Lab Technician assigns Patient Number
11. LabTech Writes Patient Number on Consents& Survey
12. Consents and Survey are collected in Results Folder
13. Lab Tech Labels 3 red top tubes with Patient Number and Date of blood draw
14. Lab Tech draws 3 red top tubes Patient Blood
15. Blood clots for 30 minutes – 1 hour
16. Lab Tech Centrifuges blood
17. LabTech labels serum tubes w/ Patient number& Date
18. Lab Tech removes serum and places it in tubes
19. Serum is placed in refrigerator before collection
20. Tubes of serum are collected daily

Okuserulriza namba ----------   Enkora 1.1   Ebiro-------/--------/----------

Form yokweikiriza okwetaba omukuseruliriza handwaire ezikwata abantu hamu nebisoro

Oyeserwe okwetaba omukuseruliriza handwaire ezikwata abantu hamu nebisoro omu Hoima district, Uganda. Okomerwemu habwokuba ozire ha irwaro linu lya grade 4 nkomurwaire rundi osendekereize omurwaire akuserura obujanjabi. Tukusaba osome kurungi ebinyakuli omu kihandiko kinu kandi okaguze kyona ekyotayetegereze otakaikirizi okwetaba omukuseruliriza kunu.

Okuseruliriza kunu kukorwa Jonathan Dyal MPH owa university ya Minnesota na Makerere university neraba omukitongole kyensi yona ekyensonga zabana. Kikusagikwa Doris Duke Charitable Trust's International Clinical Research Fellowship nibaraba omu University ya Minnesota.

Ekigenderwa kyokuseruliriza kunu

Ekigemderwa kiri kwetegereza biki ebiretereza endwaire okuruga omubisoro zikakwata abantu. Okuseruliriza kwija kuba nikuteka muno ensira hamuswija gwente na Crimean Congo-hemorrhagic fever hamu nendwire ezindi..

Emitendere yokuseruliriza kunu

Obworaba oikirize kwetaba omukuseruliriza kunu tukusaba kukora binu

- Garukamu ebikaguzo binu ebikukwatana nemyerabize yawe ekusobora kukuletera kukwatwa endwaire zebisoro nka omuswija gwente na Crimean Congo-hemorrhagic fever.
- Hayo omusahi gwawe ogukwingana sisi 12 (12mls) okuruga omukinywa hali omukebezi womusahi oweirwaro linu.

Ebizibu ebiri omukwetaba omukuseruliriza kunu

- Okusasa kuke hali baraiha omusahi
- Okuzimba kake hali baraiha omusahi
- Obundi ekitakanya kutahwa obuhuka

Amagoba agali omukwetaba omukuseruliriza kunu

- Kutunga amagezi habikukwatana nendwaire nka omuswija gwente na Crimean Congo-hemorrhagic feve.r

- Kukonyera abantu abandi hamu nabebembezi okwetegereza endwaire zinu nkoku zijanjaramu hamu nokuzetangira omubyaro.

Encwamu ezindi hakwetaba omukuseruliriza kunu.

- Obutetaba omukuseruliriza kunu.

Okusasurwa omukuseruliriza kunu.

Busaho sente zoona ezirasasura omuntu weena hakwetaba omukuseruliriza kunu.

Kakusangwa ohutara omukuseruliriza kunu

Kakusanga ohutara omukuseruliriza kunu noija kujanjabwa nohebwa obujanjabi bwokubanza (first aid) rundi obujanjabi obundi. Okusasura obujanjabi kulikorwa nkoku tukora bulijo hali ahutaire rundi insurance kampuni. Kandi kakusangwa ohutara omukuseruliriza kunu tera repota ahali akukwatwaho ahonaho.

Okwahura ensita

Ebihandiko ebikukwatagana nokuseruliriza kunu bija kwahurwa omunsita. Kakusangwa tukora ekihandiko kyona ekikukwatagana nokuseruliriza kunu titulyolekya ibara lyo omuntu weena ayayetabire omukuseruliriza kunu. Kyonka ebimuratugambira nibisobora kurabwamu aba Doris Duke Charitable Foundation’s International Clinical Research Fellowship ne bitongole ebindi ebya university niba linda ensita. Kandi busaho kintu kyona ekirahandikwa omu bihandiko byomuntu weena hairwo lyona. omukuseruliriza kunu twija kuhondera ebiragiro bya university omukukwata nokwahura ebiratugambirwa.

Okwegondeza omukuseruliriza kunu

Oli wobugabe kwetaba rundi obutetaba omukuseruliriza kunu kandi encwamu yawe teina kakwate nenkoragana yawe na university ya Minnesota rundi health center 4 enu. Bwire bwona oli wobugabe okwemereza okwetaba kwawe omukuseruliriza kunu.

Owokuhikira no kukaguza

Okuseruliriza kunu kukorwa Jonathan Dyal MPH, Dr. Katey Pelican Ph.D DVM, na Dr. Lawrence Mugisha, Ph.D DVM. Nosobora kubakaguza bwire bwona noraba ha simu zinu 0774933409.

Kakusangwa oba noyetaga kwetegereza kusingaho nosobora kutera ha Fairview Research Helpline ha namba zinu 612 -672-7692 rundi eyabusa ha 866-508-6961. Rundi nosobora kuhandikira office enu eya Dr. Jesse Kagimba, Chairman JCRC-IRB/REC, HIV/AIDS Research Committee Secretariat at Uganda National Council for Science and Technology, Plot 3 Kimera Road, 041-705-513. Oija kuhebwa kopi ya form enu.

Okweikiriza kwetaba omukuseruliriza kunu

Ekihandiko nkisomere nyakyetegereza kurungi. Nkagwize ebikaguzo nahebwa engarukamu. Hati ninyikirize kwetaba omukuseruliriza kunu. Okwikiriza kwange kukwolekwa aka box hansi na 'X' eyinteire omu ka box ako.

Nyikirize okwetaba omukuseruliriza kunu.

Zoonotic Diseases Serologic Survey Consent Form

You are invited to participate in a research study of diseases in animals which can affect humans in Hoima, Uganda. You were selected as a possible participant because you presented to the Health Center 4 today as an outpatient or are a caregiver accompanying an outpatient. We ask that you read this form and ask any questions you may have before agreeing to be in the study.

This study is being conducted by Jonathan Dyal, MPH through the University of Minnesota's Division of Global Pediatrics in collaboration with Makerere University, College of Veterinary Medicine, Animal Resources and Biosecurity. Support for this project is from the Doris Duke Charitable Trust's International Clinical Research Fellowship through the University of Minnesota.

Study Purpose

The purpose of the study is to understand the extent of and risk factors for a prior infection with diseases that pass from animals to humans. The study will be focusing on Brucellosis and Crimean Congo-hemorrhagic fever in addition to other infectious diseases.

Study Procedures

If you agree to participate in this study, we would ask you to do the following:
- Answer a short survey about your behaviors related to livestock and possible risk factors for infection with Brucellosis or Crimean Congo-Hemorrhagic Fever.
- Submit one 12mL (about 2 and a half teaspoons) whole blood sample by venipuncture taken by the clinic laboratory technician.

Risks of Study Participation

The study has the following risks.
- Pain or discomfort at the site of venipuncture.
- Bruising at the site of venipuncture.
- On extremely rare occasions, there is a small risk of infection.

Benefits of Study Participation

- There are no benefits to study participation

Alternatives to Study Participation
- Not participating in the study

Study Costs/Compensation
- No money will be given to participants in the study.

Research Related Injury

In the event that this research activity results in an injury, treatment will be available, including first aid, emergency treatment and follow-up care as needed. Care for such injuries will be billed in the ordinary manner to you or your insurance company. If you think that you have suffered a research related injury, let the study physicians know right away.

Confidentiality

The records of this study will be kept private. In any publications or presentations, we will not include any information that will make it possible to identify you as a subject. Your record for the study may, however, be reviewed by representatives from The Doris Duke Charitable Foundation's International Clinical Research Fellowship and by departments at the University with appropriate regulatory oversight. No information will be recorded in your medical record. To these extents, confidentiality is not absolute. Study data will be encrypted according to current University policy for protection of confidentiality.

Voluntary Nature of the Study

Participation in this study is voluntary. Your decision whether or not to participate in this study will not affect your current or future relations with the University or the Health Center IV. If you decide to participate, you are free to withdraw at any time without affecting those relationships.

Contacts and Questions

The researchers conducting this study are Jonathan Dyal MPH, Dr. Katey Pelican Ph.D DVM, and Dr. Lawrence Mugisha, Ph.D DVM. You may ask any questions you have now, or if you have questions later, **you are encouraged to** contact them at 0774933409.

In case of any questions regarding the Welfare and rights of participants, you should contact Dr. Jesse Kagimba, the **Chairman JCRC-IRB/REC** or the HIV/AIDS Research Committee Secretariat at Uganda National Council for Science and Technology, Plot 3 Kimera Road, Ntinda on telephone 041-705-513.

You will be given a copy of this form to keep for your records.

Statement of Consent

I have read the above information. I have asked questions and have received answers. I consent to participate in the study. My consent is indicated by drawing and "X" in the box below.

☐

I consent to participate in the Zoonotic Disease Surveillance Study.

Number_________ Sample Storage Informed Consent Date: _____/_____/_____

To Participants in the Zoonotic Disease Seroprevalence (ZDSS) Study:

By signing below, I (print name) ____ agree to have up to 12 mL of my blood and/or serum drawn as a part of the Zoonotic Disease Seroprevalence Study (ZDSS) stored for further research. I understand that the stored samples will be used for additional tests to better understand diseases that pass from animals to humans, such as, but not limited to, Crimean Congo Hemorrhagic Fever, Brucellosis, Anthrax, and Leptospirosis. I understand that my samples will be deidentified and stored at the Ugandan Ministry of Agriculture, Animal Industry and Fisheries (MAAIF) under the supervision of faculty from the Makerere University College of Veterinary Medicine, Animal Resources and Biosecurity. I consent that my samples may be used for any other research project proposed by the supervising faculty that is deemed acceptable by an Internal Review Board, without being personally contacted regarding these additional studies. I understand that storing my samples is completely voluntary, and that I can participate in ZDSS without storing my samples. I know that my decision will in no way affect the quality of medical care that I receive. I recognize that possible risks of storing my samples include:

- Discovery of additional medical information without my knowledge
- Use of my samples to develop new medical technologies or tests without my knowledge

Benefits of storing my samples include:

- Contributing to a greater understanding of other zoonotic diseases in my community
- Supporting Ugandan research initiatives and the development of new technologies

I understand that I can choose not to have my samples stored for future research without penalty or loss of benefits to which I am otherwise entitled.

In marking an X below, I acknowledge that I have read and understood the above information, and choose to have my samples obtained through the ZDSS stored for future research.

Statement of Consent

I have read the above information. I have asked questions and have received answers. I consent to have my samples stored for the study. My consent is indicated by drawing and "X" in the box below.

☐

I consent to have my samples stored for the Zoonotic Disease Surveillance Study.

Human Behavioral Risk Survey

A. Demographics

1. Sub County: ________________ 2. Village: _______________
3. Age: 0) 18-29 1)30-39 2) 40-49 3) 50-59 4) 60-69 5) 70-79 6) 80+
4. Sex: 0)MALE 1)FEMALE
5. Religion: 0)CHRISTIAN 1) MUSLIM 2)TRADITIONAL
6. Educational Level: 0) NONE 1)PRIMARY 2) SECONDARY 3) POST SECONDARY
7. Occupation (please mark all that apply):

0)HERDSMAN 1)DOMESTIC WORK 2)UNSKILLED LABOR 3)OWN BUSINESS 4)PROFESSIONAL

5)SKILLED LABOR 6)FISHING 7)BUTCHER 8)ABBATOIR 9)FARMING 10)NONE

11) OTHER: _____________

8. Time Living in Hoima: 0) 0-1 yrs 1) >1 – 3yrs 2) >3 – 5yrs 3) >5 – 10 yrs 4)10+ yrs
9. If 0-1 yrs, where did they live before coming to Hoima? 0) N/A -->#14 1) Yes, --> #10

10. Country: 11. District: 12. County: 13. Village:

B. Health History

14. In your entire life, have you ever suffered from: 0) NONE

1)MALARIA 2)TUBERCULOSIS 3)CRIMEAN-CONGO HEMORRHAGIC FEVER

4)BRUCELLOSIS 5)ANTHRAX 6)LEPTOSPIROSIS 7)TYPHOID

15. Has anyone in your village ever suffered from: 0) NONE

1)RABIES 2)CRIMEAN-CONGO FEVER 3)BRUCELLOSIS 4)ANTHRAX 5)LEPTOSPIROSIS

16. Within the past year, have you suffered from fever? 0)NO →#21 1)YES →#17

17. If yes, was a cause diagnosed? 0)NO→#21 1)YES → #18 2) Unknown→#18

18. Diagnosis 1:_________________________ 21. Diagnosis 2: ____________________

19. Treated? 0)NO 1)YES 22. Treated? 0)NO 1) YES

20. Treatment outcome? 0)CURE 1)RELAPSE 23. Outcome? 0) CURE 1)RELAPSE

2)CHRONIC INFECTION 3)NO EFFECT 2) CHRONIC INFECTION 3)NO EFFECT

Human Behavioral Risk Survey (continued)

C. Contact with Animals

How many of each livestock do you own?

24)CATTLE_____ 25)GOATS_____ 26)SHEEP_____ 27) BIRDS (chicken, duck, etc) _____

28)PIGS______ 29) DOGS______ 30)CATS_______ 31)DONKEYS__________

In the last month, how many times have you consumed the following animal products:

32)RAW MILK _____ 33)FERMENTED MILK_____ 34)SHABWE_____ 35)BOILED MILK_____

36)BEEF_____ 37)CHICKEN_____ 38)GOAT_____ 39)SHEEP_____ 40) PIG_____ 41)BLOOD_____

42) OTHER (ie: wild deer, monkey, chimpanzee) ________________

43. Which of these activities involving CATTLE have you done in the past two weeks? 0)NONE

1)HERDING 2)MILKING 3)ASSIST BIRTH 4)SLAUGHTER 5)BUTCHERING 6)SKINNING
7)OTHER:

44. Which of these wildlife and/or wild pests live near your home? 0) NONE

1)MONKEYS 2)BABOONS 3)CHIMPANZEES 4)RATS 5)BATS 6)WILD DEER

7)OTHER (ie: carnivores, elephants, hippo) 41.Please specify-

45. How often do you visit forested areas? 0) NEVER 1) DAILY 2) WEEKLY
3)MONTHLY 4) LESS THAN MONTHLY

46. When do you visit forested areas? 0)NEVER 1)MORNING 2)DAYTIME 3)EVENING

47. In the last year, how many ticks have bitten you? __________

D. Domestic Environment

48. Type of housing: 0)MUD & THATCH 1)MUD, COW DUNG & THATCH
2) MUD & IRON 3)CONCRETE AND IRON

49. Drinking Water sources: 0)HOME PIPES 1) BORE HOLE 2)WELL 3)PUBLIC TAP
4)SURFACE WATER
